# Leptospirosis: An Unusual Cause of ARDS

**DOI:** 10.1155/2010/408365

**Published:** 2010-05-26

**Authors:** Marc Clavel, Gwenaelle Lhéritier, Nicolas Weinbreck, Antoine Guerlin, Anthony Dugard, Eric Denes, Philippe Vignon

**Affiliations:** ^1^Medical-Surgical Intensive Care, Dupuytren University Hospital, Limoges, France; ^2^Department of Pathology, Dupuytren University Hospital, Limoges, France; ^3^Department of Thoracic Surgery, Dupuytren University Hospital, Limoges, France; ^4^Department of Infectious Diseases, Dupuytren University Hospital, Limoges, France

## Abstract

Severe leptospirosis usually associates shock, jaundice, renal failure, and thrombocytopenia. Massive hemoptysis due to diffuse alveolar haemorrhage may rarely occur leading to an acute respiratory failure and multiple organ failure. We present the case of an acute respiratory distress syndrome caused by a severe leptospirosis. The severity of the respiratory failure contrasted with the absence of significant liver or renal dysfunction. Bedside open lung biopsy was only consistent with a postinfectious BOOP. The diagnosis was retrospective when the niece of the patient presented with similar inaugural symptoms ten days later after being scratched by a wild rat which was considered by our patient as a pet.

## 1. Introduction

We report a case of a cannabis and ectasy user with multiple episode of hemoptysis, persistent hypoxia and bilateral lung infiltrates corresponding to the definition of an acute respiratory distress syndrome (ARDS). The final diagnosis was subsequently established by another “case contact”.

## 2. Case Report

**Figure 1:**
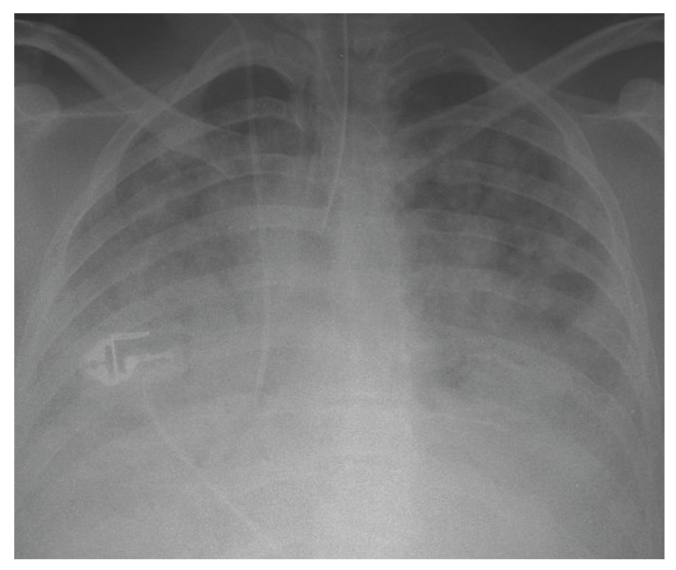
Postero anterior radiograph of the chest obtained after tracheal intubation on day 1.

**Figure 2:**
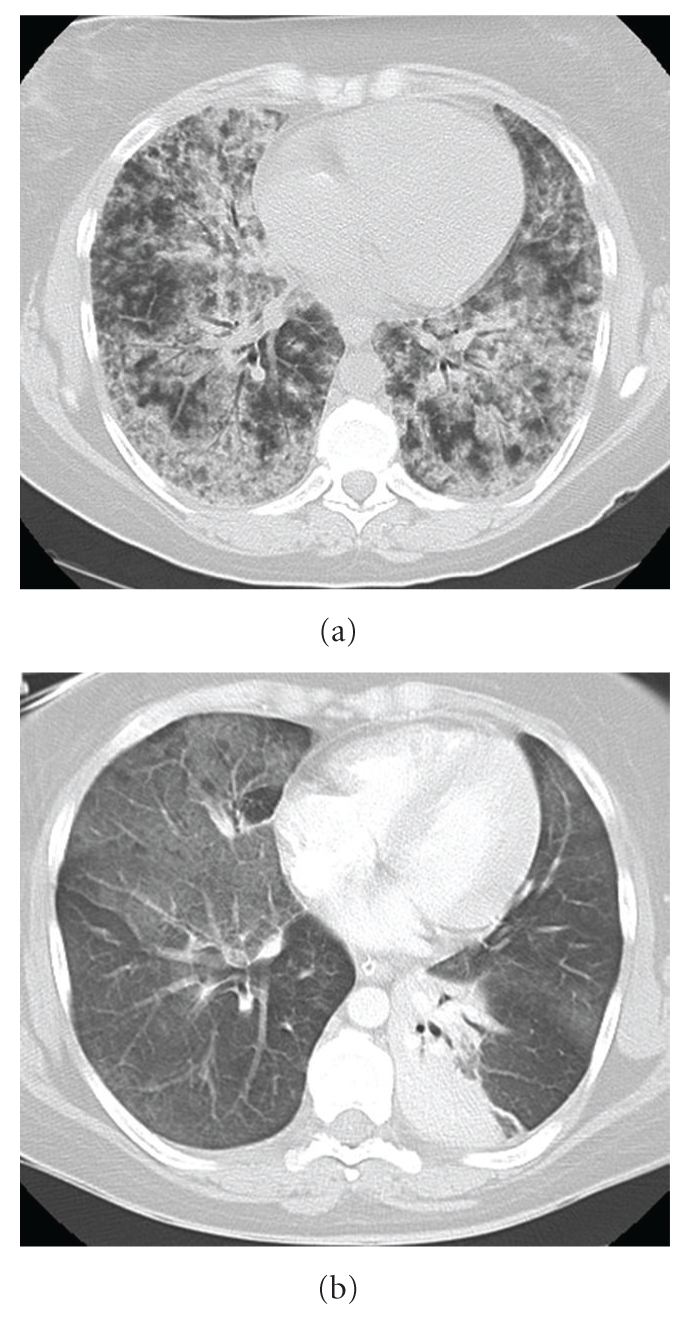
(a) CT scan of the chest obtained on day 1 showing diffuse alveolar infiltrate. (b) CT scan of the chest obtained on day 8: right ground glas opacities and atelectasis of the left lower lobe.

A 30-year-old woman who was a current cannabis and ectasy user presented with hemoptysis and acute respiratory distress. She also complained of fever, nausea, vomiting, myalgias and headache since 5 days. Her medical history was only significant for acute pancreatitis secondary to alcohol abuse. Physical examination confirmed high body temperature (40.5°C), normal blood pressure and urine output and crackles were present in lower lobes of both lungs. Laboratory results were as follows: WBC count, 7.4 × 10^3^/*μ*L; platelets, 97 × 10^3^/*μ*L; hematocrit, 27%; C reactive protein, 231 mg/L; BUN, 3.4 mmol/L; creatinine, 61 *μ*mol/L, aspartate transaminase, 43 U/L (normal <32 U/L); alanine transaminase, 37 U/L (normal <32 U/L); alkaline phosphatase, 112 U/L (normal <104 U/L) and total bilirubin, 15 mg/dL, with a direct component of 5 mg/dL. Arterial blood gas measurements (on mask with reservoir bag 15 l/min oxygen) were as follows: pH, 7.48; PaCO_2_, 30 mm Hg; PaO_2_/FiO_2_ = 52, base excess, −1.5 mmol/L. Admission chest X-ray showed diffuse bilateral infiltrates (Figure 1). A CT scan of the chest on day 1 demonstrated diffuse alveolar infiltrates more pronounced in the lower lobes (Figure 2(a)). She received ceftriaxone and levofloxacine.

**Figure 3:**
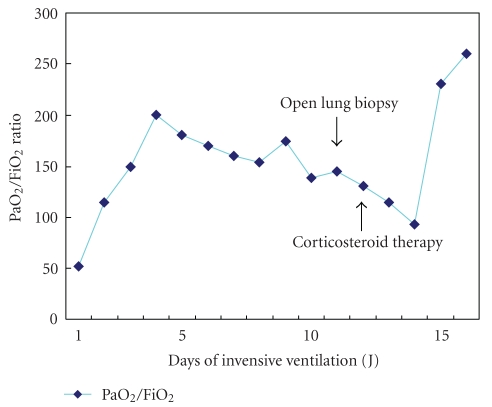
PaO_2_/FiO_2_ ratio during invasive ventilation.

**Figure 4:**
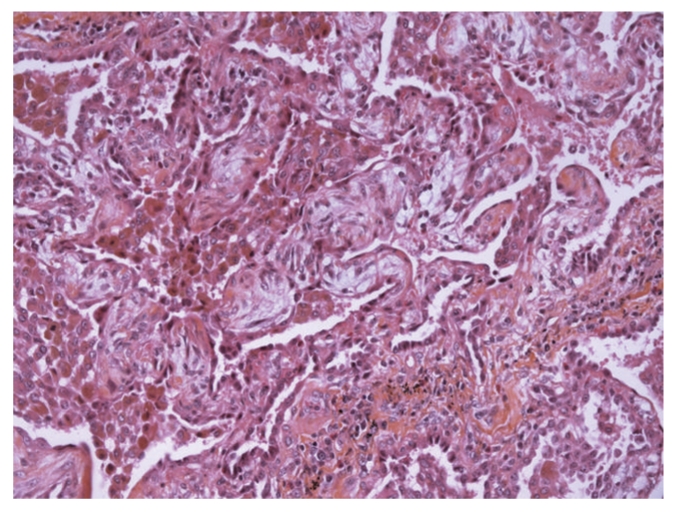
Bedside open lung biopsy performed on day 9 (HES × 200): lesions of bronchiolitis obliterans with organizing fibroblastic polyps in alveoli.

The patient had recurrent hemoptysis (300 ml total) and required intubation for hypoxemia. A ventilatory support with lung protective strategy was instituted (initial settings: assist-control mode, FiO_2_ = 1, Peep = 10 cm H_2_O, inspiratory flow 60 l/min, tidal volume 6 ml/kg, mean airway pressure = 27 cm H20). A transoesophageal echocardiography showed a normal left ventricular function with low filling pressures. The patient underwent a bronchoscopy with BAL. Bacterial, mycobacterial, viral and fungal culture findings of the fluid were negative. Significant hemoptysis persisted during 4 days. HIV serology was negative. Another CT scan of the chest performed on day 8 showed a marked regression of alveolar infiltrates but the presence of ground-glass opacities (Figure 2(b)). An open-lung biopsy was performed on day 9 due to persistent hypoxemia despite protective ventilation (Figure 3). Pathological analysis pointed out typical lesions of bronchiolitis obliterans with organizing fibroblastic polyps in bronchioles, alveolar ducts and alveoli but without inflammatory cells (Figure 4). The patient received prednisolone (1 mg/kg) from day 10. Due to rapid improvement of her respiratory condition, she was extubated on day 15. Patient's niece presented to our institution on day 12 with headache, fever, myalgias and meningismus. She declared having being scratched by a wild rat domesticated by her aunt. The initial serology for leptospirosis was negative, the second determination three weeks after was clearly positive (MAT positive at 1 : 3200 for *Leptospiro icterohaemorrhagiae).* Results were also positive for the niece.

## 3. Discussion

In spite of a classic inaugural clinical presentation, the absence of hepatic and renal involvement led to the delayed diagnosis of leptospirosis. Although frequent, pulmonary symptoms are usually mild in leptospirosis [1]. Severe form of leptospirosis including major hemorrhagic complications is usually associated with jaundice and renal impairment (*icteric leptospirosis)*. In a cohort of 26 Spanish patients, 7 patients presented hemoptysis and only 3 patients had an ARDS but always associated with a multi organ failure [2]. Our case report underlines that ARDS can be the manifestation of the disease even in the absence of significant renal or hepatic involvement. An ARDS requiring mechanical ventilation is associated with a mortality rate as high as 30 to 60% [3]. To controlling the bleeding, platelet transfusions and desmopressin therapy proved to be effective in massive pulmonary hemorrhage in patients with severe leptospirosis [4]. A recent publication mentions the interest of cyclophosphamide in patients with leptospiral pulmonary alveolar hemorrhage: out of the 33 patients treated with cyclophosphamide, 22 (66.7%) survived, while in the control group out of 32 patients, three (9.4%) survived. The hypothesis is that an exacerbated immune response of the host plays an important role in its pathogenesis [5]. In our patient a vascularitis and Goodpasture's disease have been first ruled out. Unusual causes of ARDS have been systematically checked: a “crack lung” was suspected because of patient's medical history, but recent use of cocaine was denied by patient's relatives and the research in her hairs was negative. Mycoplasma pneumoniae infection was excluded by a negative test for Mycoplama pneumoniae IgM and a negative cold agglutinin test. An herpes virus infection was also pushed aside. The ARDS seems to be specific and directly triggered by leptospires or by their antigenic products affecting endothelial cells of pulmonary capillaries. We respected a lung protective strategy in the management of the ARDS associated with restrict fluid intake. The prone position 16 hours a day was performed during the first days but without significant improvement of oxygenation status. The absence of diffuse alveolar damage on lung biopsy can be interpreted as an early recovery or as a new example of discordance between American European Consensus Conference clinical criteria of ARDS and histopathological findings [6]. The histological findings of BOOP led us to initiate a corticosteroid therapy which facilitated a dramatic improvement of patient respiratory status. This case confirms the value and the safety of bedside open lung biopsy in patients with refractory ventilated ARDS [7]. The use of the corticosteroid therapy was already described as well in the management of pulmonary leptospirosis as in the ARDS. In a series of 30 patients, Shenoy et al. demonstrated that corticosteroids reduce mortality and change outcome significantly [8]. In the same way, the works in favor of a premature utilisation of corticosteroid in ARDS multiply [9]. We hypothesized that the patient was cured by the empiric antibiotherapy with ceftriaxone

Leptospirosis is a zoonosis caused by spirochetes from the species *Leptospira interrogans. *The illness is transmitted to humans by indirect or direct contact with infected animals. Nardone et al. isolated the risk factors for leptospirosis in metropolitan France: skin lesions, canoeing, contact with wild rodents and a home in the countryside [10].

To conclude, severe leptospirosis may lead to ARDS in the absence of hepatic or renal dysfunction. Clinicians have to be aware of pulmonary forms and risk factors for leptospirosis.

##  Conflicts of Interests

The authors declared that they have no conflicts of interests.

## Abbreviations

BAL: Bronchoalveolar lavage
ARDS: Acute respiratory distress syndrome
BOOP: Bronchiolitis obliterans organizing pneumonia.
